# Perception of and attitude toward ethical issues among Korean occupational physicians

**DOI:** 10.1186/s40557-017-0182-z

**Published:** 2017-06-24

**Authors:** Junghye Choi, Chunhui Suh, Jong-Tae Lee, Segyeong Lee, Chae-Kwan Lee, Gyeong-Jin Lee, Taekjoong Kim, Byung-Chul Son, Jeong-Ho Kim, Kunhyung Kim, Dae Hwan Kim, Ji Young Ryu

**Affiliations:** 1 Department of Occupational and Environmental Medicine & Institute of Environmental and Occupational Medicine, Busan Paik Hospital, Inje University, Busan Paik hospital, 75, Bokji-ro, Busanjin-gu, Busan, South Korea; 2 Department of Medical Humanities, Inje University, Busan, South Korea; 3 Department of Humanities and Social Sciences in Medicine, Inje University College of Medicine & Institute for Medical Humanities, Inje University, Busan, South Korea; 4 Department of Occupational and Environmental Medicine, Haeundae Paik Hospital, Inje University, Busan, South Korea

**Keywords:** Core professional values, Ethical code, Ethical conflicts, Ethical issues, Ethical principles, Occupational physician

## Abstract

**Background:**

Occupational physicians (OPs) have complex relationships with employees, employers, and the general public. OPs may have simultaneous obligations towards third parties, which can lead to variable conflicts of interests. Among the various studies of ethical issues related to OPs, few have focused on the Korean OPs. The aim of the present survey was to investigate the ethical contexts, the practical resolutions, and the ethical principles for the Korean OPs.

**Methods:**

An email with a self-administered questionnaire was sent to members of the Korean Society of Occupational and Environmental Medicine, comprising 150 specialists and 130 residents. The questionnaire was also distributed to 52 specialists and 46 residents who attended the annual meeting of the Korean Association of Occupational and Environmental Clinics in October 2015, and to 240 specialists by uploading the questionnaire to the online community ‘oem-doctors’ in February 2016. The responses to each question (perception of general ethical conflicts, recognition of various ethical codes for OPs, core professional values in ethics of occupational medicine, and a mock case study) were compared between specialists and residents by the chi-squared test and Fisher’s exact test.

**Results:**

Responses were received from 80 specialists and 71 residents. Most participants had experienced ethical conflicts at work and felt the need for systematic education and training. OPs suffered the most ethical conflicts in decisions regarding occupational health examination and evaluation for work relatedness. Over 60% of total participants were unaware of the ethical codes of other countries. Participants thought ‘consideration of worker’s health and safety’ (26.0%) and ‘neutrality’ (24.7%) as the prominent ethical values in professionality ofoccupational medicine. In mock cases, participants chose beneficence and justice for fitness for work and confidential information acquired while on duty, and beneficence and respect for autonomy in pre-placement examinations.

**Conclusions:**

This study evaluated the current perception of and attitude toward ethical issues among the Korean OPs. These findings will facilitate the development of a code of ethics and the ethical decision-making program forthe Korean OPs.

**Electronic supplementary material:**

The online version of this article (doi:10.1186/s40557-017-0182-z) contains supplementary material, which is available to authorized users.

## Background

Ethics is a generic term dealing with diverse ways of considering and understanding moral life. Morality refers to a system of ideas with regard to right and wrong human conduct that is so widely shared, albeit incomplete, that it constitutes a stable social agreement. Professional morality is a particular type of morality that includes moral codes of practice. The ethics of a profession comprises the obligations determined by the accepted role of professionals [1].

As occupational health (OH) professionals, the role of occupational physicians (OPs) is to protect and promote workers’ health and working ability; this role varies among countries depending on national legislation and the relationships among employers, employees, and the general public [2, 3]. Responsibilities to these parties may conflict. For example, ethical problems are most likely to arise if one party does not perceive that the OPs have other responsibilities [4]. Some ethical codes have been produced by professional societies and organizations for OPs [4–8], and provide guidance on what is considered right or wrong in terms of practice [9]. However, these do not cover the ethical conflicts facing OPs in Korea, due to the limited ability to extend specific approaches beyond general ethics to consider Korean contextual issues.

Despite this, there have been few studies on the necessity of an ethical code for Korean OPs with respect to the ethical conflicts faced, solutions employed, knowledge of the ethical code, and attitude toward implementation of ethical principles. Only one survey of 107 Korean OPs on perception of ethical issues during their practice was conducted in 2010. OH practice is constantly changing and OPs need guidelines to address new ethical challenges. Therefore, this study aimed to investigate the ethical conflicts, the practical resolutions, the ethical principles, and core professional values for the Korean OPs.

## Methods

### Study population and survey method

A total of 661 specialists and 130 residents were registered in the Korean Society of Occupational and Environmental Medicine on February 16, 2015. Among them, the email addresses of 150 specialists and 130 residents were obtained from academic activity by residents, several alumni associations of Departments of Occupational and Environmental Medicine in Busan, and the excerpts of the annual meeting of the Korean Society of Occupational and Environmental Medicine in 2015. We mailed the questionnaire to these individuals. One month later, we sent a follow-up request to non-responders to the first mailing, and a second follow-up request 1 month later to non-responders to the second request. All questionnaires were analyzed within 2 months from the date of the last mailing. During the survey period (October 2015 to February 2016), 41 specialists and 45 residents replied. Also, questionnaires were obtained from 38 specialists and 26 residents among 52 specialists and 46 residents who attended the annual meeting of the Korean Association of Occupational and Environmental Clinics in October 2015. Furthermore, we received two responses from specialists who were members of the online community ‘oem-doctors,’ which comprised 9 fourth-year residents and 240 specialists in occupational and environmental medicine, during February 2016. We analyzed the survey data of 80 specialists and 71 residents. One specialist did not complete the questionnaire.

### Questionnaire design

An eight-page self-administered questionnaire with 17 questions was provided to all OPs in the study. The questionnaire consisted of four main parts: participants’ general characteristics, perception of general ethical conflicts and recognition of various ethical codes, priorities in 10 core professional values, and mock case studies involving ethical conflicts in OH practice.

The first part included: age, gender, years of expertise, grade of residency, workplace (university hospital, general hospital, local clinic, research institute, and others), and the main role in the OH practice (occupational health examination, industrial healthcare management in small and medium enterprises (SMEs), occupational research, company doctor, and others). The second part comprised four questions about ethical conflicts in the participant’s OH practice: 1) the frequency, 2) the object, 3) the area of the ethical conflicts, and 4) resources for a solution; and three questions about the requirement for ethical decision-making: 1) the necessity of systematic ethical training, 2) need for guidelines on ethics for OPs, and 3) the level of sense of ethics required by OPs. The participants were also asked whether they had heard of the following ethical codes of OH practice; the code of ethical conduct published by the American College of Occupational and Environmental Medicine (ACOEM) [6], the International Code of Ethics for Occupational Health Professionals of the International Commission on Occupational Health (ICOH) [5], the Guidance on Ethics for Occupational Physicians by the UK Faculty of Occupational Medicine [7], and the Code of Ethics for the Practice of Industrial Hygiene by the American Conference of Governmental Industrial Hygienists (ACGIH) [8].

In the third part, participants listed 10 core professional values for ethics in OH practice. These 10 items were developed by Kim [10] based on the Code of Ethical Conduct published by the ACOEM, ICOH Code of Ethics. Participants were asked to rank the items in the order of most to least important. The item considered most important was given a rank of 1, the next most important a rank of 2 and so on. Responses were analyzed by counting only a rank of 1 in each of the items, because we reasoned that most respondents would have difficulty ranking more than three items at a time, as reported by Reetoo [11].

To determine which of four moral principles (respect for autonomy, nonmaleficence, beneficence, and justice) was preferred for resolution of ethical conflicts in OH practice, eight mock cases were presented in the fourth part of the questionnaire (Additional file 1). A series of expert group meetings were held in person and via email to discuss current and emerging topics in OH practice. These groups comprised specialists in occupational environmental medicine and medical humanities, as well as the author team. Cases were developed based on these discussions and on the studies of Kim [10], Martimo [12], and Brandt-Rauf [13]. The eight mock cases covered three topics: fitness for work (cases 1, 4, and 5), pre-placement examination (cases 2 and 6), and confidential information acquired while on duty (cases 3, 7, and 8). The participants were asked to choose one of the four choices, which were combinations of four moral principles; two specialists in medical humanities reviewed the choices in each of the cases. Finally, an open-ended question was included in the last part of the questionnaire to evaluate the types of ethical conflict experienced by participants.

### Statistical analysis

The responses to all questions were compared between specialists and residents. The second part of the questionnaire regarding the perception of general ethical conflicts was analyzed within each group according to the number of years of expertise for specialists and the grade of residency for residents. The chi-squared test and Fisher’s exact test were used to evaluate differences between groups. Statistical significance was assessed using IBM SPSS Statistics 21.0 for Windows (IBM Corp., Armonk, NY, USA). A *P*-value of <0.05 was considered to indicate statistical significance.

## Results

### Characteristics of participants

A total of 151 responses, including 80 specialists and 71 residents, were analyzed. The general characteristics of respondents are shown in Table 1. The mean age of participants was 38.3 ± 8.7 years (44.0 ± 7.9 years for specialists and 32.0 ± 3.7 years for residents). Specialists were 82.5% male and 17.5% female, and residents were 84.5% male and 15.5% female. The distribution by workplace of specialists was 33.8% university hospital, 22.5% general hospital, 25.0% local clinic, 7.5% research institute, 3.8% company, and 2.5% public health center for serving a military duty. The major roles were in OH examination (68.8%), followed by industrial healthcare management in SMEs (13.8%), research (5.0%), and company doctor (3.8%). The mean number of years of expertise was 10.4 ± 6.5, with a minimum of 1.0 and a maximum of 22.0. The distribution grade of residents was 32.4% first year, 25.4% second year, 18.3% third year, and 23.9% fourth year.

**Table 1:** General characteristics of the subjects

Participant characteristic	Specialist (*N* = 80)	Resident (*N* = 71)
	n (%)	n (%)
Age		
20–29	0 (0)	22 (31.0)
30–39	30 (37.5)	47 (66.2)
40–49	25 (31.3)	2 (2.8)
50–59	22 (27.5)	0 (0)
≥ 60	3 (3.8)	0 (0)
Gender		
Male	66 (82.5)	60 (84.5)
Female	14 (17.5)	11 (15.5)
Work site ^a^		
University hospital	27 (33.8)	
General hospital	18 (22.5)	
Local clinic	20 (25.0)	
Research institute	6 (7.5)	
Others	9 (11.3)	
Main role ^a^		
Occupational health examination	55 (68.8)	
Industrial healthcare management in the SME^b^	11 (13.8)	
Occupational research	4 (5.0)	
Company doctor	3 (3.8)	
Others	7 (8.8)	
Years of expertise		
≤ 10 years	45 (56.3)	
> 10 years	35 (43.8)	
Grade of residency		
1st-year		23 (32.4)
2nd-year		18 (25.4)
3rd-year		13 (18.3)
4th-year		17 (23.9)

^a^Not investigated for residents in terms of work site and main role

^b^ *SME* small- and medium-sized enterprises

### Perception of general ethical conflicts

In this study, 91.3% of specialists and 63.4% of residents responded that they had experienced ethical conflicts during their practice; the difference was significant. There was no significant difference according to years of expertise of specialists or grade of residents. Moreover, 87.5% of specialists and 71.8% of residents responded that systematic education and training in ethical decision-making were necessary. Specialists had experienced a greater number of ethical conflicts than residents. Most specialists believed that systemic ethical training was necessary, irrespective of years of expertise. However, a greater proportion of senior residents (third and fourth years) compared with junior residents (first and second years) agreed with the need for such training, but the difference was not significant. Of specialists and residents, 76.3% and 59.2%, respectively, considered themselves to have a higher sense of ethics than physicians of other specialties; the difference was significant. Of specialists and residents, 81.0% and 76.1%, respectively, responded to the question regarding the need for a code of ethics for OPs. Specialists stated that they had experienced ethical conflicts in making decisions during OH examinations (43.6%), work relatedness (27.0%), and health management (15.4%). Other responses were assessment of fitness for work (7.7%) and epidemiologic surveys (3.8%). Of the residents, 46.5% experienced ethical conflicts in evaluation of work relatedness and 38% in OH examinations. The object of ethical conflicts experienced by both specialists and residents was in the order employee and employer. To resolve ethical conflicts, 47.2% of specialists and 26.8% of residents used their personal beliefs, and 40.3% of specialists and 54.9% of residents consulted with physician colleagues. Only 2% of specialists and residents relied on an ethical code (Table 2).

**Table 2:** Responses to questions on the perception of general ethical conflicts

Item	All subjects		*P* value	Specialist		*P* value	Resident		*P* value
	Specialist	Resident		〉10 years	≤10 years		3rd & 4th-year	1st & 2nd-year	
	(*N* = 80)	(*N* = 71)		(*N* = 35)	(*N* = 45)		(*N* = 30)	(*N* = 41)	
	n (%)	n (%)		n (%)	n (%)		n (%)	n (%)	
Have you ever struggled with ethical problems during work?			0.000			0.693*			0.322
Yes	73 (91.3)	45 (63.4)		31 (88.6)	42 (93.3)		21 (70.0)	24 (58.5)	
No	7 (8.7)	26 (36.6)		4 (11.4)	3 (6.7)		9 (30.0)	17 (41.5)	
Have you ever felt the necessity of systematic education and training regarding ethical decision-making during work?			0.016			0.5*			0.065
Yes	70 (87.5)	51 (71.8)		32 (91.4)	38 (84.4)		25 (83.3)	26 (63.4)	
No	10 (12.5)	20 (28.2)		3 (8.6)	7 (15.6)		5 (16.7)	15 (36.6)	
Do you think that an occupational physician has to have a higher sense of ethics than any other doctor?			0.024			0.487			0.271
Yes	61 (76.3)	42 (59.2)		28 (80.0)	33 (73.3)		20 (66.7)	22 (53.7)	
No	19 (23.7)	29 (40.8)		7 (20.0)	12 (26.7)		10 (33.3)	19 (46.3)	
Do you think it is necessary to have an ethical code for occupational physicians? ^a^			0.459			0.745			0.918
Yes	64 (81.0)	54 (76.1)		29 (82.9)	35 (80.0)		23 (76.7)	31 (75.6)	
No	15 (19.0)	17 (23.9)		6 (17.1)	9 (20.0)		7 (23.3)	10 (24.4)	
What is the area with the most frequent ethical conflict on your work? ^b^									
Decision of general health examination	0 (0)	4 (5.6)		0 (0)	0 (0)		0 (0)	4 (9.8)	
Decision of occupational health examination	34 (43.6)	27 (38.0)		17 (50.0)	17 (38.6)		12 (40.0)	15 (36.5)	
Evaluation for work relatedness	21 (27.0)	33 (46.5)		9 (26.4)	12 (27.3)		15 (50.0)	18 (43.9)	
Industrial healthcare management	12 (15.4)	2 (2.9)		4 (11.8)	8 (18.2)		2 (6.7)	0 (0)	
Etc.	11 (14.0)	5 (7.0)		4 (11.8)	7 (15.9)		1 (3.3)	4 (9.8)	
What is the most frequent object of ethical conflict on your work?									
Employee	32 (40.0)	34 (47.9)		13 (37.1)	19 (42.2)		16 (53.3)	18 (43.9)	
Employer	31 (38.8)	18 (25.4)		15 (42.9)	16 (35.6)		12 (40.1)	6 (14.6)	
Government	1 (1.1)	2 (2.7)		1 (2.9)	0 (0)		0 (0)	2 (4.9)	
Doctor colleague	4 (5.0)	6 (8.5)		1 (2.9)	3 (6.7)		1 (3.3)	5 (12.2)	
Paramedics	3 (3.8)	3 (4.2)		1 (2.9)	2 (4.4)		1 (3.3)	2 (4.9)	
Etc.	9 (11.3)	8 (11.3)		4 (11.3)	5 (11.1)		0 (0)	8 (19.5)	
How do you resolve an ethical conflict that occurs at work? ^c^									
Consult with other doctor colleagues	29 (40.3)	39 (54.9)		8 (25.8)	21 (51.2)		12 (40.0)	27 (65.9)	
Resolve with personal belief	34 (47.2)	19 (26.8)		17 (54.8)	17 (41.5)		8 (26.7)	11 (26.8)	
Consult with healthcare specialist	3 (4.2)	5 (7.0)		3 (9.7)	0 (0)		4 (13.3)	1 (2.4)	
Consult with the chief of the related department	0 (0)	6 (8.5)		0 (0)	0 (0)		6 (20.0)	0 (0)	
Refer to the ethical code	2 (2.7)	2 (2.8)		2 (6.4)	0 (0)		0 (0)	2 (4.9)	
Etc.	4 (5.6)	0 (0)		1 (3.3)	3 (7.3)		0 (0)	0 (0)	

*P-values by Fisher’s exact test (other *p*-values were calculated by chi-squared test)

^a^One non-responding specialist with ≤10 years’ experience was excluded from the analysis

^b^One multiple-responding specialist with ≤10 years’ experience and one multiple-responding specialist with >10 years’ experience were excluded from the analysis

^c^Four non-responding specialists with ≤10 years’ experience, three non-responding specialists >10 years’ experience, and one multiple-responding specialist with >10 years’ experience were excluded from the analysis

### Recognition of various ethical codes for OPs

Participants were permitted to provide multiple responses to awareness of world-renowned ethical codes in OH practice (Table 3). In total, 37 specialists (46.2%) and 21 residents (29.6%) were aware of the ACOEM Code of ethical conduct, the ICOH Code of Ethics, the UK Faculty of Occupational Medicine Code of Ethics, the ACGIH Code of Ethics, and other ethical codes. The percentage of subjects who were aware of each code was as follows: ICOH Code of Ethics 22.5%, ACOEM Code of Ethical Conduct 21.9%, ACGIH Code of Ethics 5.3%, the UK Faculty of Occupational Medicine Code of Ethics 3%, and other ethical codes 3%. One specialist and one resident were aware of all four ethical codes. Ninety-three (61.6%) of the 151 participants were not aware of the above codes. Kim [10] in 2010 reported that 55.1% of respondents were unaware of the codes; therefore, awareness of ethical codes has not increased over the past 6 years.

**Table 3:** Recognition of various ethical codes by occupational physicians

Ethical codes	Specialist	Resident	Total	Kim’s Total [10]
	(*N* = 80)	(*N* = 71)	(*N* = 151)	(*N* = 107)
	n (%)	n (%)	n (%)	n (%)
ACOEM ^a^ [6]	24 (30)	9 (12.7)	33 (21.9)	29 (22.5)
ICOH ^b^ [5]	16 (20)	18 (25.4)	34 (22.5)	20 (15.5)
FOM ^c^ [7]	2 (2.5)	1 (1.4)	3 (2)	5 (3.9)
ACGIH ^d^ [8]	4 (5)	4 (5.6)	8 (5.3)	15 (11.6)
Others	3 (3.8)	0 (0)	3 (2)	1 (0.8)
I have never heard of that.	43 (53.8)	50 (70.4)	93 (61.6)	59 (55.1)

^a^ACOEM: American College of Occupational & Environmental Medicine

^b^ICOH: International Commission on Occupational Health

^c^FOM: Faculty of Occupational Medicine

^d^ACGIH: American Conference of Governmental Industrial Hygienists

### Core professional values in ethics of occupational medicine

The four most important characteristics regarding professional values for ethics of occupational medicine were ‘consideration of workers’ health and safety’ (26.0%), ‘neutrality’ (24.7%), ‘effort to maintain expertise as an OP’ (20.0%), and ‘maintenance of professional independence by excluding unwarranted interference from third parties such as employers and labor unions’ (14.0%). Specialists chose ‘consideration of workers’ health and safety’ (31.6%) as the most important professional value, while residents selected ‘neutrality’ (35.2%). Of the specialists with >10 years’ experience, 22.9% and 14.4% selected ‘neutrality’ and ‘maintenance of professional independence by excluding unwarranted interference from third parties’, respectively, compared with 9.1% and 20.4%, respectively, for specialists with ≤10 years’ experience. In contrast, Kim [10] reported that responses were in the order of ‘neutrality’ (32.7%), ‘effort to maintain expertise’ (24.3%), ‘consideration of workers’ health and safety’ (20.6%), and ‘maintenance of professional independence by excluding unwarranted interference from third parties such as employers and labor unions’ (10.3%). In this study, the four most important characteristics were identical but their rank order differed (Table 4).

**Table 4:** Core professional values related to the ethics of occupational medicine

Professional value	All subjects		Specialist		Resident			
	Specialist	Resident	>10 years	≤10 years ^a^	3rd & 4th-year	1st & 2nd-year	Total	Kim’s Total [10]
	(*N* = 79)	(*N* = 71)	(*N* = 35)	(*N* = 44)	(*N* = 30)	(*N* = 41)	(*N* = 150)	(*N* = 107)
	n(%)	n(%)	n(%)	n(%)	n(%)	n(%)	n(%)	n(%)
Consideration of worker’s health and safety	25 (31.6)	14 (19.7)	10 (28.6)	15 (34.1)	7 (23.2)	7 (17.1)	39 (26.0)	22 (20.6)
Neutrality	12 (15.2)	25 (35.2)	8 (22.9)	4 (9.1)	11 (36.7)	14 (34.1)	37 (24.7)	35 (32.7)
Efforts to maintain expertise	17 (21.5)	13 (18.3)	6 (17.1)	11 (25.0)	6 (20.0)	7 (17.1)	30 (20.0)	26 (24.3)
Maintenance of professional independence	14 (17.7)	7 (9.9)	5 (14.4)	9 (20.4)	2 (6.7)	5 (12.2)	21 (14.0)	11 (10.3)
Confidentiality	4 (5.1)	8 (11.3)	3 (8.6)	1 (2.3)	2 (6.7)	6 (14.6)	12 (8.0)	3 (2.8)
No discriminative attitude for individual worker	3 (3.8)	2 (2.8)	1 (2.8)	2 (4.5)	0 (0)	2 (4.9)	5 (3.3)	1 (0.9)
Efforts to understand and improve a harmful environment in the workplace	2 (2.5)	2 (2.8)	1 (2.8)	1 (2.3)	2 (6.7)	0 (0)	4 (2.8)	6 (5.6)
Heath education to improve an occupational hazard and worker’s bad heath habits	1 (1.3)	0 (0)	1 (2.8)	0 (0)	0 (0)	0 (0)	1 (0.6)	0 (0)
Worker’s complete understanding and consent during a medical examination or the investigation of a work environment	1 (1.3)	0 (0)	0 (0)	1 (2.3)	0 (0)	0 (0)	1 (0.6)	3 (2.8)
Maintenance of cooperative relationship with a health administrator in the company	0 (0)	0 (0)	0 (0)	0 (0)	0 (0)	0 (0)	0 (0)	0 (0)

^a^ One specialist with ≤10 years’ experience did not respond and was excluded from the analysis

### Mock case study

In response to eight mock cases of ethical conflicts in OH practice, OPs selected one of four moral principles: respect for autonomy, nonmaleficence, beneficence, and justice (Table 5). In the second case, specialists considered the principles of respect for autonomy (61.3%) and beneficence (30.0%) to be important, while the residents considered respect for autonomy (56.3%), beneficence (21.1%), and nonmaleficence (12.7%) to be important. In the other cases, the responses of the specialists and residents were similar.

**Table 5:** Distribution of preferred moral principles in mock cases^a^

	Moral principles				*P* value
	Beneficence	Nonmaleficence	Respect for autonomy	Justice	
Case 1					0.426
Total (*N* = 147)	44 (29.1)	3 (2.0)	4 (2.6)	96 (63.6)	
Specialist (*N* = 76)	26 (32.5)	1 (1.3)	3 (3.8)	46 (57.5)	
Resident (*N* = 71)	18 (25.4)	2 (2.8)	1 (1.4)	50 (70.4)	
Case 2					0.025*
Total (*N* = 148)	39 (25.8)	10 (6.6)	89 (58.9)	10 (6.6)	
Specialist (*N* = 78)	24 (30.0)	1 (1.3)	49 (61.3)	4 (5.0)	
Resident (*N* = 70)	15 (21.1)	9 (12.7)	40 (56.3)	6 (8.5)	
Case 3					0.934
Total (*N* = 148)	33 (21.9)	23 (15.2)	7 (4.6)	85 (56.3)	
Specialist (*N* = 77)	16 (20.0)	13 (16.3)	4 (5.0)	44 (55.0)	
Resident (*N* = 71)	17 (23.9)	10 (14.1)	3 (4.2)	41 (57.7)	
Case 4					0.298
Total (*N* = 149)	97 (64.2)	19 (12.6)	24 (15.9)	9 (6.0)	
Specialist (*N* = 78)	52 (65.0)	10 (12.5)	14 (17.5)	2 (2.5)	
Resident (*N* = 71)	45 (63.4)	9 (12.7)	10 (14.1)	7 (9.9)	
Case 5					0.499
Total (*N* = 150)	126 (83.4)	12 (7.9)	6 (4.0)	6 (4.0)	
Specialist (*N* = 79)	68 (85.0)	4 (5.0)	4 (5.0)	3 (3.8)	
Resident (*N* = 71)	58 (81.7)	8 (11.3)	2 (2.8)	3 (4.2)	
Case 6					0.701
Total (*N* = 150)	99 (65.6)	1 (0.7)	37 (24.5)	13 (8.6)	
Specialist (*N* = 79)	51 (63.8)	1 (1.3)	21 (26.3)	6 (7.5)	
Resident (*N* = 71)	48 (67.6)	0 (0)	16 (22.5)	7 (9.9)	
Case 7					0.279
Total (*N* = 150)	75 (49.7)	39 (25.8)	4 (2.6)	32 (21.2)	
Specialist (*N* = 79)	43 (53.8)	22 (27.5)	2 (2.5)	12 (15.0)	
Resident (*N* = 71)	32 (45.1)	17 (23.9)	2 (2.8)	20 (28.2)	
Case 8					0.328
Total (*N* = 148)	118 (78.1)	17 (11.3)	10 (6.6)	3 (2.0)	
Specialist (*N* = 77)	65 (81.3)	7 (8.8)	3 (3.8)	2 (2.5)	
Resident (*N* = 71)	53 (74.6)	10 (14.1)	7 (9.9)	1 (1.4)	

^a^Nonresponse and multiple responses were excluded from the analysis

Case 1 involved assessment of fitness for work of an individual undergoing peritoneal dialysis, who travels to rural areas on business. Of the participants, 63.6% chose the principle of justice, in which asking the individual to resign was considered excessive, and instead, recommended department transfer to reduce the workload; and 29.1% chose beneficence, which allowed the individual to work after adjusting work intensity as it would be helpful for he/she to work in the interest of disease control. Three specialists who did not respond and one specialist who provided multiple responses were excluded from this analysis. Case 2 involved a worker with a long smoking history who gains a job that involves exposure to asbestos. Of the participants, 58.9% (61.3% of specialists and 56.3% of residents) responded that the person could work with protective equipment after appropriate training, based on the individual’s opinion. The remaining 30% of specialists chose beneficence in terms of permitting the individual to take up employment after quitting smoking, provided that he/she has regular health checkups. However, 21.1% of residents chose beneficence while 12.7% of residents chose nonmaleficence, in terms of prohibiting the person from doing the job in question. Two specialists who did not respond and one specialist who provided multiple responses were excluded from the analysis. Case 3 was related to the discovery of a new health hazard; 56.3% of participants chose justice, in terms of attempting to convince the employer of the social issue by accumulating related data, and 21.9% chose beneficence in terms of reporting the issue to the authorities to achieve improvement. Two specialists who did not respond and one specialist who provided multiple responses were excluded from the analysis. Case 4 focused on the ethical justification of assessments of fitness for work, in which 64.2% of participants chose beneficence; i.e., to benefit the health of the worker. Of the participants, 15.9% chose respect for autonomy in terms of information provision to workers. Finally, 12.6% of participants chose nonmaleficence, i.e., prevention of harm. Two non-responding specialists were excluded from the analysis. Case 5 involved the fitness for work of a worker with a mental condition. Of the participants, 80% chose beneficence in the form of work transition, except for work requiring communication with co-workers. One non-responding specialist was excluded from the analysis. Case 6 had to do with a worker with diabetes undergoing pre-placement examination for a job in an assembly plant. Of the participants, 65.6% chose beneficence in terms of granting permission to work under the condition of blood sugar control and annual health checkups, while 24.5% chose respect for autonomy by granting permission to work with consideration of the worker’s opinion, as there were insufficient data linking carpal tunnel syndrome to diabetes. One non-responding specialist was excluded from the analysis. Case 7 involved a company in which the OP was responsible for health management and was asked to remain silent regarding products containing carcinogenic asbestos. Of the participants, 49.7% chose beneficence in terms of not allowing workers or consumers to use the product, while 25.8% chose the principle of nonmaleficence in terms of forcing the employer to improve the work environment and suspend use of the product. Other participants (21.2%) chose justice in terms of blocking product distribution due to excessive healthcare costs. One specialist who selected all three options was excluded from the analysis. Case 8 had to do with an employer who refused to examine the suggested link between a suspicious substance and occupational disease. Most participants (78.1%) chose beneficence in terms of evaluating the risk. Two specialists who did not respond and one specialist who provided multiple responses were excluded from the analysis.

## Discussion

Medical professionalism is the promise and duty of a professional group of physicians to fulfill social expectations based on a social contract, and can also be a belief in the need for professional behavior under social regulation [14]. A society expects the medical profession to fulfill their guarantee of competence, altruism, morality, truthfulness, promotion of public benefit, transparency, accountability, and self-regulation, as opposed to granting autonomy and providing exclusive rights. Because the medical profession is responsible for duties necessary to society, the standard required is high both practically and morally [15, 16]. In other words, medical professionalism is based on expertise and ethics and on the premise that professional autonomy as a physician exists within a professional standard and code of ethics [17]. Because OPs have complicated relationships with employees, employers, and the general public, they may have simultaneous obligations towards a third party, which could lead to adverse effects and ethical conflicts [18]. In this context, ethical consideration during OH practice is regarded as an important core competency for OPs [2, 11, 19, 20]. As a result, many countries establish and improve their own ethical code in accordance with their circumstances [4, 6–8].

This study showed that Korean OPs, 91.3% of specialists and 63.4% of residents (70% of senior residents), experienced many work-related ethical conflicts; these percentages are likely due to differences in levels of authority and responsibility between specialists and residents. Many respondents experienced ethical conflicts with employees and employers during OH examinations and evaluations of fitness for work. Korean OPs conduct OH examinations and industrial healthcare management in SMEs based on the Occupational Safety and Health Acts [21]; as such, Korean OPs are closely connected with the Ministry of Health and Welfare and the Ministry of Employment and Labor. Moreover, as their customers are commonly not patients but healthy employees and most OPs are hired instead of being in private practice, decision-making processes usually involve consultation with all parties concerned [22]. Many respondents claimed that they experienced ethical conflicts with related parties during decision-making for work-related diseases (especially with workers diagnosed with an occupational disease, D1). Also specialists had more frequent conflicts with employers than residents, likely because specialists have more responsibilities and contact with employers, such as in industrial healthcare management and OH examinations. Specialists resolve such conflicts through their personal beliefs and consultation with colleagues, while residents mostly do so with colleagues; few respondents used available ethical codes. This differed from the studies by Brandt-Rauf’s in American OPs, in which 22% of respondents ‘always’ and 33% ‘frequently’ resolved ethical conflicts using professional codes of ethics [13]. Thus, professional codes of ethics can assist in resolution of ethical conflicts during OH practice. In this study, 81% of specialists and 76.1% of residents recognized the necessity for a code of ethics for OPs in Korea, particularly those more experienced. Additionally, those with more experience also tended to more frequently agree that systematic training for ethical decision-making and a higher sense of ethics are needed for OPs, compared with other physicians.

Most of the participants experienced ethical conflicts at work, but few were aware of other codes of ethics for OPs. The level of recognition was not significantly different from that of a survey performed in 2010 [10]. This may be because many Korean OPs resolve ethical conflicts based on their personal beliefs and consultation with an experienced colleague. However, the study by Aw of recognition of ethical codes involving OPs from the UK, the Netherlands, and Singapore reported that all but one of the physicians were unaware of codes formulated by organizations outside of their own country [9]; it is thus possible that Korean OPs believe that ethical codes from other countries are not relevant to the Korean context. Also, compared with national guidelines targeting OPs in the country of origin, such as the Guidance on Ethics for Occupational Physicians by the UK Faculty of Occupational Medicine [7] or the Code of Ethics for the Practice of Industrial Hygiene by ACGIH in the United States [8], the International Code of Ethics for Occupational Health Professionals by the ICOH, which targets international OPs [5], was recognized by only 20% of the participants in this study. This may be because the general description and instructions for each ethical principle are inadequate to resolve ethical conflicts in practice. Indeed, the American Medical Association Code of Medical Ethics sets out in detail the ethical and legal problems physicians may face [23], and the British Medical Association’s Medical Ethics Today provides a detailed explanation and common cases of ethical problems [24]. Therefore, a suitable Korean ethical code for OPs is required.

The four core professional values, ‘consideration of workers’ health and safety’, ‘neutrality’, ‘effort to maintain expertise’ and ‘maintenance of professional independence’, did not differ from the configuration items in Kim’s study [10], except for ranking; specifically, OPs in Korea regarded the following to be important obligations: 1) to protect worker’s health, respect their human dignity, and maintain their well-being; 2) to judge based on scientific knowledge and fact, and refrain from any judgment and action against integrity and impartiality; 3) to continue to develop competence, be aware of scientific/technical knowledge, and aim to achieve the best recognized standard of quality; and 4) not be affected by a conflict of interest in any situation during judgment and decision-making [5, 25, 26].

Compared with Martimo’s study, in which ‘expertise’ and ‘confidentiality’ were selected as the core ethical characteristics of OH, our results differed [12]. Confidentiality, a key factor in several ethical codes [4–8], is defined as the protection of medical data. Disclosure and transmission of medical information should be controlled by national laws or regulations and ethical codes for medical practitioners [5]. Confidentiality is supposed to be strictly guaranteed in OH practice; however, it is not an absolute principle [27]. OPs have dual (or multiple) loyalties; thus, they are put in the position of assessing the risk-benefit of all parties concerned. As such, it is inevitable that OPs are concerned over what and how much information should be provided. Korean OPs judged confidentiality to be of low importance in this study, possibly due to cultural differences. A specialist responded to an open-ended question that he experienced a conflict with the confidentiality of workers’ medical information when he reported to the employer. Thus, more attention should be paid to confidentiality.

Residents and specialists with >10 years’ experience selected neutrality as their first and second choices, respectively. However, neutrality was ranked fourth by specialists with ≤10 years’ experience. This could reflect their priorities but could also be due to confusion over the concepts of neutrality and independence. A limitation of this study was that these terms were not defined in the survey.

The mock cases enable identification of the ethical principles used by Korean OPs in practice. The four-principle (beneficence, nonmaleficence, respect for autonomy, and justice) approach to biomedical ethics by Beauchamp and Childress is widely accepted in current biomedical theory [28]. The principles of nonmaleficence (to do no harm to others) and beneficence (to provide benefit) have been important in medical ethics from ancient times. However, the principles of respect for autonomy (the right of self-determination) and justice (fair distribution of benefit, risk, and cost) have emerged since the mid-twentieth century. This is because decision-making has been progressively transferred from the physician to the patient, and the distribution of medical resources has become an important issue [1, 15].

The principle of beneficence and justice were selected for assessment of fitness for work (cases 1, 4, and 5) and confidential information acquired while on duty (cases 3, 7, and 8). As assessment of fitness for work aims to ensure that workers are in an occupational environment appropriate for their psychological and physical capability; OPs judge whether a worker’s health is suitable. Ethical conflicts can occur in this process because the worker and employer may have different perspectives. OPs can also experience ethical conflicts due to their having multiple loyalties. For example, OPs have revealed that a hazardous material has been used or an event has been harmful to the health of the worker or the general public. In this study, many OPs acted from the perspective of beneficence, especially paternalism, which is intentional intervention in another person’s preference or actions with the purpose of either preventing or reducing harm to or benefiting that person. This was because Korean OPs already operate within a social context that takes good deeds for workers and public health for granted.

In terms of a pre-placement examination, beneficence was preferred in case 6, and respect for autonomy in case 2. Pre-placement examination is an assessment of fitness for work based on the health condition of the individual, the job description, and harmful factors [29]. There can be a conflict between the right to work (respect for autonomy) and health (beneficence). In case 2, both specialists (61.3%) and residents (56.3%) chose respect for autonomy as the preferred ethical principle. As the second choice, beneficence (30.0%) was chosen by specialists, and beneficence (21.1%) and nonmaleficence (12.7%) by residents. Nonmaleficence is a passive duty to do no harm to others, while beneficence actively promotes and preserves health [15]. The difference is likely due to the different responsibilities of specialists and residents.

This is the first study of supplication of four moral principles to resolve ethical conflicts. According to Westerholm, it is necessary to clarify the facts needed for a decision, to identify which of the ethical values mentioned above are involved, and to confirm whose interests are concerned and the consequences of an action by the OP [30]. This study revealed the preferred ethical values of Korean OPs. Further studies are required to assess the contributions and limits of the ethical principles.

Some of the items can be understood differently due to the absence of ethical training for Ops and ambiguity of the ethical terms between ‘neutrality’ and ‘maintenance of professional independence’. The numerator of the response rate could be identified because information on the name, age, year acquired (for specialists), and grade of residency (for residents) were available. However, we were unable to confirm the denominator of total contacted persons, due to the three surveys used, personal information protection, and the potential for duplication among the three survey methods (e.g., sending an email to several alumni associations through administrators, attendance at the annual autumn meeting of Korean Association of Occupational and Environmental Clinics, and survey via the online community). The inability to calculate the response rate is a weakness of this study. However, this approach enabled collection of a more diverse range of opinions.

## Conclusions

This study identified the current perception of and attitudes toward ethical issues among Korean OPs. Most participants had experienced ethical conflicts at work, especially for decisions regarding occupational health examination and evaluation for work relatedness. ‘Consideration of worker’s health and safety’ and ‘neutrality’ were identified as the core professional values in OM. These findings will enable development of training programs for ethical decision-making and a code of ethics for Korean OPs.

## Additional file

Additional file 1: Mock cases. (DOCX 17 kb)

## Abbreviations

ACGIH: The American Conference of Governmental Industrial Hygienist
ACOEM: The American College of Occupational and Environmental Medicine
ICOH: The International Commission on Occupational Health
OH: Occupational health
OPs: Occupational physicians.
