# Supplementary material for: Perception of and attitude toward ethical issues among Korean occupational physicians
Source: Ann Occup Environ Med. 2017 Jun 24;29:23. doi: 10.1186/s40557-017-0182-z (PMC5482971; doi:10.1186/s40557-017-0182-z)
Supplement: Additional file 1: — Mock cases. (DOCX 17 kb) [file 40557_2017_182_MOESM1_ESM.docx]

**Appendix 1. Mock cases**

Case 1

A 47-year-old man, Mr. A, periodically went on business trips to remote areas. He had recently undergone peritoneal dialysis surgery after being diagnosed with nephrotic syndrome. His department manager concluded that it was unsafe for him to work and reported this to the employer, even though Mr. A wished to continue working while on self-peritoneal dialysis. The employer considered department transfer or urging Mr. A to resign, and then asked your opinion about his periodic business trips to remote areas. What would you do?

① Respect the worker’s willingness to go on business trips to remote areas, and report that he is able to fulfill this duty.

② Because the patient is on peritoneal dialysis, suggest that it would be difficult for him to continue work.
③ Under the assumption that regular working hours will assist disease control, suggest that he is able to work after adjustment of the working conditions (intensity etc.).

④ Viewing resignation as excessive, instead suggest that he be transferred to another department.

Case 2

A 50-year-old man visits for a pre-placement examination. There is nothing unusual except a long smoking history of 50 pack-years. He plans to work as a demolition contractor, where he would be exposed to asbestos. What would you do?

① Prohibit him from working at the company, due to the risk of asbestos exposure.

② Allow him to work after stopping smoking immediately, and suggest regular health examinations.

③ Respect his willingness to work, and allow him to work for the company while wearing personal protective equipment at all times.

④ Prevent him from working for the company if the lung cancer management cost of a smoker exposed to asbestos negatively affects the company’s employee health management.

Case 3

Your company introduced new chemicals to improve productivity. Soon thereafter, it was found that workers who manage the chemicals have an increased frequency of a certain disease. A subsequent investigation and literature review indicated a relationship between the new chemicals and the disease. The employer requested that you remain silent about the relationship. What would you do?

① Notify the workers to resolve the problem themselves.

② Notify the labor union to urge the employer to improve the working environment and stop using the chemicals responsible.
③ Contact the authorities immediately to achieve improvement.
④ Accumulate more data to convince the employer of a social issue.

Case 4

Assessment of fitness for work in occupational health examinations may limit career opportunities by identifying those at high risk. Why are assessments of fitness for work ethically justified?

① Occupational medicine is a medically professionalized sector concerned with workers’ health, life, and benefits.
② As OPs, it is important to prevent dangerous situations and injury.
③ Assessments can prevent healthcare resource allocation problems due to occupational diseases.
④ It is important to provide to workers information regarding their health.

Case 5

A 35-year-old man, Mr. A, repeatedly took up and quit work due to somatoform disorder several years ago. Two years ago, he was employed as a technician, which involved frequent communication with co-workers. However, his job performance deteriorated and he had severe conflicts with his co-workers. Therefore, he was re-evaluated after taking 1 month of medical leave. A psychiatrist reported that his mental status is stable, and Mr. A was willing to return to work. However, the plant supervisor reported to the employer that it was unsafe for Mr. A to return to work. The employer considered department transfer or urging Mr. A to resign, and then sought your opinion. What would you do?

① Respect the worker’s willingness to work, and report that he is fit to return to work.

② Report that his mental status is such that he may be a threat to his co-workers and/or himself.

③ Under the assumption that regular working will benefit his health, suggest that Mr. A is fit to work in another department as he is currently mentally stable.

④ Notify the company to manage his worksite and working conditions only, and suggest that the worker undergo treatment at his own expense.

Case 6

A 40-year-old woman, Ms. B, visited for a pre-placement examination. Ms. B was diagnosed with diabetes, and her new job was in an assembly plant and involved simple repetitive tasks using the hands and wrists. Companies in similar industries had experienced decreased productivity as a result of carpal tunnel syndrome, so employers tried to find ways to prevent it. Simple repetitive tasks can cause carpel tunnel syndrome, as can diabetes. However, no reliable data show that the risk of carpal tunnel syndrome is higher in employees with diabetes. What would you do?

① If diabetes is a possible cause of carpal tunnel syndrome, prohibit Ms. B from working for the company.

② Allow her to work for the company after checking her diabetes control, and suggest annual health checkups.

③ Due to the lack of data regarding the effect of diabetes on the risk of carpal tunnel syndrome, allow Ms. B to work for the company in order to respect her willingness.
④ If the management cost of the incidence of carpal tunnel syndrome in persons with diabetes does not negatively affect the company’s employee health management, Ms. B can be hired.

Case 7

After reviewing the material safety data sheet of an adhesive used in the company in which you are responsible for industrial healthcare management, you find that it contains a large amount of asbestos. The owner immediately discarded the adhesive and replaced it with a safe alternative, but did not take any further action and requested that you remain silent to prevent product recall and/or litigation. What would you do?

① Take no further action if workers are willing to work.

② Notify the owner of the potential risk to health, suggest that the owner perform a work environment evaluation and improvement, and discontinue use of the manufactured products.

③ Suggest discontinuation of use of products manufactured using the adhesive by both workers and consumers.

④ The management costs of the health problem due to use of the product may be excessive, so prohibit distribution of the products.

Case 8

A large number of patients suspected to have occupational bronchial asthma were identified during an occupational health examination of a company. After verifying the substance that caused the disease, you notified the supervisor that it was necessary to perform additional investigations and take appropriate actions. However, he requested you to report only the result of the occupational health examination, as the substance involved was not in the occupational health examination category and no further investigations were needed. What would you do?

① Take no further action if employees are willing to work.

② Report only the result related to the occupational health examinations, as the substance is not in the examination category and therefore you cannot determine whether it is harmful.
③ Perform additional investigations because the harmful substance should be reported, even though it does not fall in the examination category.
④ Report only related results as the examination procedure is already structuralized with proper reason, and further examination represents a waste of resources.
